# Transplantation of MHC-mismatched mouse embryonic stem cell-derived thymic epithelial progenitors and MHC-matched bone marrow prevents autoimmune diabetes

**DOI:** 10.1186/s13287-019-1347-1

**Published:** 2019-08-06

**Authors:** Min Su, Yujun Lin, Zhixu He, Laijun Lai

**Affiliations:** 1 Department of Human Histology and Embryology, School of Basic Medical Sciences, Stem Cell and Tissue Engineering Research Centre, Guizhou Medical University, Guiyang, Guizhou China; 2 Department of Allied Health Sciences, University of Connecticut, 1390 Storrs Road, Storrs, CT 06269 USA; 3 Key Laboratory for Adult Stem Cell Translational Research, Chinese Academy of Medical Sciences, Guiyang, 550004 China; 4 Department of Pediatrics, Affiliated Hospital of Zunyi Medical College, Zunyi, China; 5 University of Connecticut Stem Cell Institute, University of Connecticut, Storrs, CT USA

**Keywords:** Type 1 diabetes, Embryonic stem cells, Thymic epithelial cells, Autoreactive T cells, Regulatory T cells

## Abstract

**Background:**

Type 1 diabetes (T1D) is an autoimmune disease resulting from the destruction of insulin-secreting islet β cells by autoreactive T cells. Non-obese diabetic (NOD) mice are the widely used animal model for human T1D. Autoimmunity in NOD mice is associated with particular major histocompatibility complex (MHC) loci and impaired islet autoantigen expression and/or presentation in the thymus, which results in defects in both central and peripheral tolerance. It has been reported that induction of mixed chimerism with MHC-mismatched, but not MHC-matched donor bone marrow (BM) transplants prevents the development T1D in NOD mice. We have reported that mouse embryonic stem cells (mESCs) can be selectively induced in vitro to generate thymic epithelial progenitors (TEPs) that further develop into thymic epithelial cells (TECs) in vivo to support T cell development.

**Methods:**

To determine whether transplantation of MHC-mismatched mESC-TEPs could prevent the development of insulitis and T1D, NOD mice were conditioned and injected with MHC-mismatched B6 mESC-TEPs and MHC-matched BM from H-2^g7^ B6 mice. The mice were monitored for T1D development. The pancreas, spleen, BM, and thymus were then harvested from the mice for evaluation of T1D, insulitis, chimerism levels, and T cells.

**Results:**

Transplantation of MHC-mismatched mESC-TEPs and MHC-matched donor BM prevented insulitis and T1D development in NOD mice. This was associated with higher expression of proinsulin 2, a key islet autoantigen in the mESC-TECs, and an increased number of regulatory T cells.

**Conclusions:**

Our results suggest that embryonic stem cell-derived TEPs may offer a new approach to control T1D.

**Electronic supplementary material:**

The online version of this article (10.1186/s13287-019-1347-1) contains supplementary material, which is available to authorized users.

## Background

Type 1 diabetes (T1D) is an autoimmune disease caused by the destruction of insulin-secreting islet β cells by autoreactive T cells [1–4]. Non-obese diabetic (NOD) mice, the most commonly used animal model for human T1D, share many of the disease characteristics including genetic risk factors, autoantigens, and the chronicity of autoimmunity [5]. Although many T1D genetic risk factors have been identified [6], the major histocompatibility complex (MHC) or human leukocyte antigen (HLA) is the major factor [1, 3]. The disease susceptibility in NOD mice or T1D patients is linked to particular MHC or HLA such as I-Ag7 or HLA-DR4 and HLA-DQ8 [7, 8]. The I-Ag7 has been shown to be unstable, and a poor peptide binder [9, 10]. This “functional instability” could lead to a defect in thymic deletion of autoreactive T cells [9–12]. Insufficient presentation of self-antigen in the thymus can also affect the development of the antigen-specific regulatory T cells (Tregs) [3].

Transgenic expression of diabetes-protective MHC alleles in the thymus has been reported to prevent T1D development in NOD mice [13, 14]. However, this approach cannot readily be translated to humans. Induction of either mixed or complete chimerism with MHC-mismatched nonautoimmune donor BM transplants also prevented the development of T1D in NOD mice [15–21]. In contrast, induction of mixed chimerism with MHC-matched nonautoimmune donor BM transplants did not prevent the development of T1D in NOD mice [19, 21]. However, in clinical settings, allogeneic BM transplantation (BMT) has the risk of inducing graft-versus-host disease (GVHD) [22].

Another strong T1D genetic risk factor is associated with a variable number of tandem repeat minisatellites, upstream of the insulin gene that modulates thymic (but not pancreatic) expression of this antigen [1]. Insulin/proinsulin is a key autoantigen in initiating anti-islet autoimmunity [23–25]. In humans, the variant that has a shorter stretch of repeats has a lower expression of insulin in the thymus, and predisposes to T1D [26, 27]. NOD mice with a deletion of the insulin 2 gene have an accelerated T1D development [28]. In contrast, transgenic expression of insulin in the thymus induces immune tolerance to the autoantigen and prevents T1D development [23]. Intrathymic injection of insulin also has the potential to prevent T1D development [29, 30]. However, to achieve this tolerance, the thymus, the major organ implicated in self-tolerance induction, has to be sufficiently functional. It is well known that the thymus undergoes age-dependent involution, and its functions are seriously compromised in the elderly [31].

T cell development occurs in the thymus, and is critically dependent on the thymic microenvironment, in which thymic epithelial cells (TECs) are the major component. However, TECs undergo both qualitative and quantitative loss over time, which is the major factor for age-dependent thymic involution. We have reported that mouse embryonic stem cells (ESCs) (mESCs) can be selectively induced to generate thymic epithelial progenitors (TEPs) in vitro [32]. When transplanted into mice, the mESC-TEPs further develop into TECs, reconstitute normal thymic architecture, and support T cell development [32, 33]. We have also shown that transplantation of mESC-TEPs expressing self-antigen myelin oligodendrocyte glycoprotein (MOG) in mice results in immune tolerance to the MOG and the prevention of experimental autoimmune encephalomyelitis (EAE) development [34, 35]. Furthermore, transplantation of donor-origin mESC-TEPs into GVHD recipients induces immune tolerance to both donor and host antigens and prevents the development of chronic GVHD [36].

In this study, we determined the ability of mESC-TEPs to prevent T1D. We show here that transplantation of MHC-mismatched C57BL/6 (B6) mESC-TEPs and MHC-matched nonautoimmune donor BM prevents insulitis and T1D development in NOD mice that were pre-conditioned with anti-CD3/CD8 antibodies (Abs). This is associated with establishing diabetes-protective MHC alleles, increasing the expression of proinsulin 2 (Ins2) in the thymus, and improving the thymic microenvironment, thereby the restoration of defective negative selection to insulin in NOD mice. In addition, transplantation of the mESC-TEPs leads to an increased number and function of Tregs in the thymus and the spleen.

## Methods

### Mice

Female NOD/LtJ, B6, and H-2^g7^ B6 mice were purchased from The Jackson Laboratory (Bar Harbor, ME). All experimental procedures involving mice were approved by the University of Connecticut Animal Care and Use Committee and were conducted in accordance with NIH guidelines.

### mESC culture and differentiation

To induce the differentiation of mESCs into TEPs, B6 mESC and GFP^+^ B6 mESC lines (from Cyagen, Santa Clara, CA) were first differentiated into definitive endoderm (DE), and then TEPs as described [36].

### Immunomagnetic cell separation

Single-cell suspensions from differentiated mESCs were harvested after the cells were treated with 2 mg/ml collagenase IV. The cells were stained with PE-labeled anti-mouse EpCAM1 antibody, washed, and stained with anti-PE MicroBeads (Miltenyi Biotec, Auburn, CA). EpCAM1^+^ and EpCAM1^−^ cells were selected using a magnetic-activated cell sorter immunomagnetic separation system (Miltenyi Biotec). Similarly, depletion of CD4^+^ cells from splenocytes, and isolation of CD45^−^EpCAM^+^ TECs were performed with immunomagnetic separation.

### Induction of mixed chimerism in NOD mice

Six-week-old NOD mice were conditioned with sequential injection of FcR- nonbinding anti-CD3ɛ F(ab’) fragment and FcR binding (FB) anti-CD3 (clone 145-2C11, from BioXCell) in addition to anti-CD8 (clone 116–13.1, from BioXCell) Abs as described [19, 21]. On day 0, the mice were injected i.v. with BM and CD4^+^ T-depleted spleen cells from MHC-mismatched B6 (H-2b) or MHC-matched congenic H-2 g7 B6 mice (20 × 10^6^ each). Some of the mice were injected intrathymically (i.t.) with mESC-TEPs (EpCAM1^+^ cells, 5 × 10^4^) or mESC-control cells (EpCAM1^−^ cells, 5 × 10^4^) on day 0 with a procedure as described [37].

### Flow cytometry analysis

Single-cell suspensions of the thymus, spleen, graft, and mESC-derived cells were stained with the fluorochrome-conjugated Abs as described [32, 33, 37, 38]. For intracellular staining, the cells were first permeabilized with a BD Cytofix/Cytoperm solution for 20 min at 4 °C. Direct or indirect staining of fluorochrome-conjugated Abs included: CD4, CD8, CD3, CD25, Foxp3, CD45, Ly51, and EpCAM1 (BioLegend or BD Biosciences, San Diego, CA), keratin (k)5 (SantaCruz Biotechnology, Santa Cruz, CA), k8 (US Biological, Swampscott, MA), fluorescein isothiocyanate, or phycoerythrin labeled anti-rat, or rabbit IgG (BD Biosciences). The samples were analyzed with an LSRFortessa X-20 Cell Analyzer (BD Biosciences). Data analysis was done using FlowJo software (Ashland, OR).

### Determination of suppressive activity of Tregs

Effector T cells (Teffs) (CD4^+^CD25^−^) and Tregs (CD4^+^CD25^+^) were purified from NOD mice by an immunomagnetic system (Miltenyi, Auburn, CA), and the purity of the cells was usually > 90%. The Teffs were stimulated with anti-CD3 antibody (Biolegend) in the presence of Tregs at a ratio of 2: 1. The proliferation of T cells was assessed by pulsing the culture with [^3^H] thymidine (1 μCi/well) (PerkinElmer, Inc., Downers Grove, IL) 12 h before harvest. Incorporation of [^3^H] thymidine was measured by liquid scintillation spectroscopy (PerkinElmer, Inc.). The suppression index = [proliferation (evaluated by CPM) without Tregs − CPM with Tregs]/CPM without Tregs.

### Real-time quantitative reverse-transcription and polymerase chain reactions (qRT-PCR)

Total RNA was isolated from cells, and cDNA was synthesized as described [36]. qRT-PCR was performed with the Power SYBR green mastermix (Applied Biosystems, UK) using the 7500 real-time PCR system (Applied Biosystems, UK).

### Statistical analysis

*P* values were based on the two-sided Student’s *t* test. A confidence level above 95% (*p* < 0.05) was determined to be significant.

## Results

### Induction of mixed chimerism with MHC-mismatched but not matched BM transplants prevents insulitis and T1D development in NOD mice

It has been reported that BM cells in combination with donor CD8^+^ T cells induced stable permanent mixed chimerism without GVHD [17]. Dr. Zeng’s group has also reported that induction of mixed chimerism with MHC-mismatched but not matched BM transplants prevents insulitis and T1D development in NOD mice pre-conditioned with anti-CD3/CD8 Abs [19]. We have used similar protocols reported by this group. Six-week-old NOD mice (H-2k^d^, I-A^g7^, CD45.1) were conditioned with anti-CD3 and anti-CD8 Abs on days − 8 and − 3. On day 0, the mice were injected i.v. with BM and CD4^+^ T cell-depleted spleen cells (20 × 10^6^ each) from MHC-mismatched B6 (H-2k^b^, I-A^b^, CD45.2) or MHC-matched congenic H-2^g7^ B6 (H-2k^d^, I-A^g7^, CD45.2) mice. The control mice were given a conditioning regimen only.

**Fig. 1:**
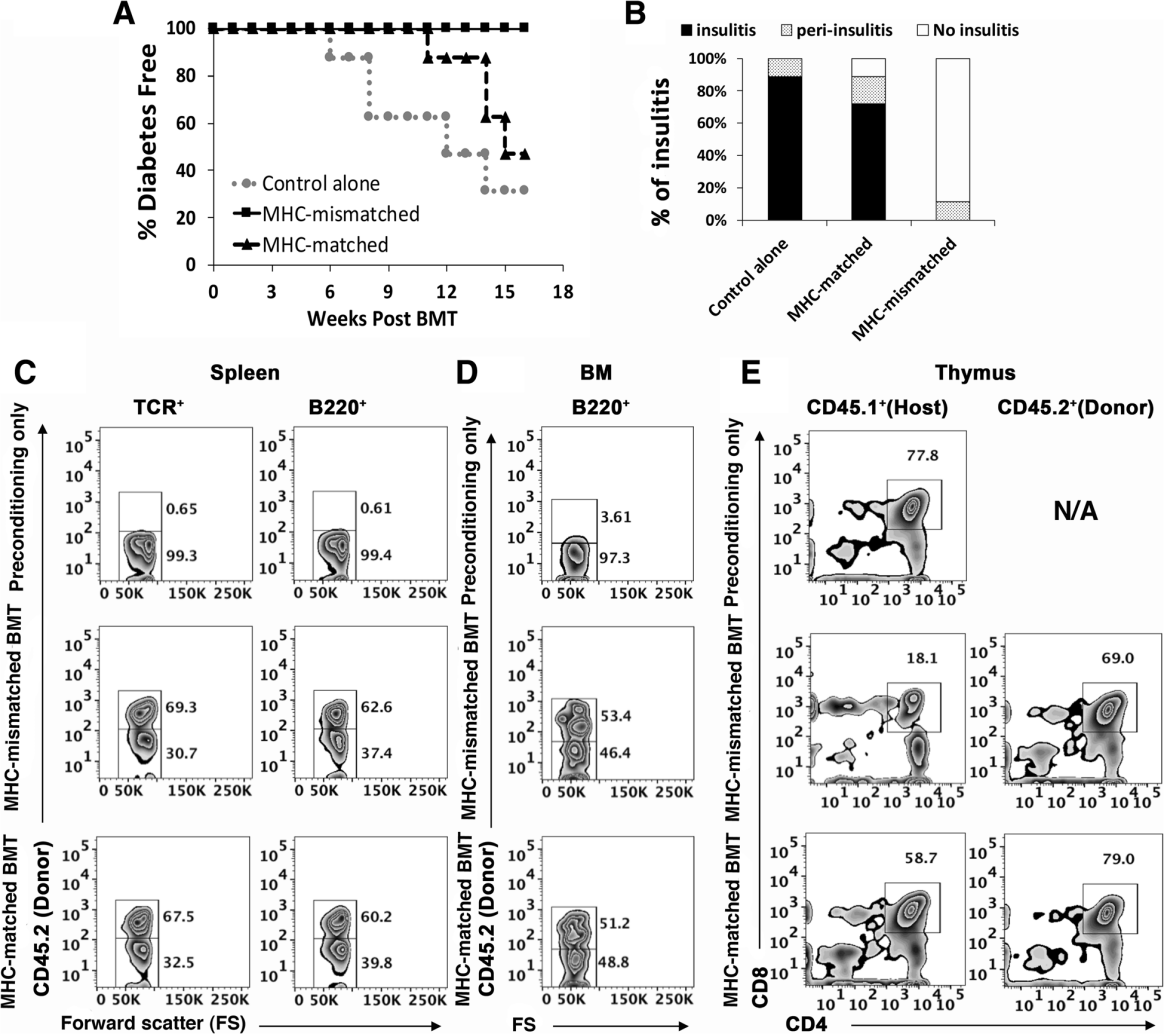
Mixed chimerism with MHC-mismatched, but not MHC-matched BM transplants prevents T1D in NOD mice. Wild-type NOD mice were injected i.v. with anti-CD3/CD8 Abs on days − 8 and − 3. On day 0, the conditioned mice were injected i.v. with CD4^+^ T cell-depleted spleen cells (20 × 10^6^ each) from MHC-mismatched B6 or MHC-matched H-2^g7^ B6 mice. The control mice were given anti-CD3/CD8 conditioning only. Diabetes development was monitored by blood glucose analysis for up to 100 days after BMT. On day 100, the pancreas, spleen, BM, and thymus were harvested from the mice. **a** Diabetes development curve after BMT. **b** Statistical analysis of the percentages of insulitis. **a**, **b** The data were pooled from three independent experiments (4–5 mice per group in each experiment). **c** Representative FACS profile of spleen cells showing the percentages of donor (CD45.2^+^) or host (CD45.2^−^) T cells (TCR^+^) and B cells (B220^+^). **d** Representative FACS profile of BM cells showing the percentages of donor (CD45.2^+^) or host (CD45.2^−^) B cells (B220^+^). **e** Gated donor (CD45.2^+^) or host (CD45.1^+^) thymocytes were shown in CD4 versus CD8. The percentages of CD4^+^CD8^+^ DP thymocytes are shown

The mice were then monitored for T1D development by analyzing blood glucose levels. On day 100, the mice were euthanized and the blood, pancreas, spleen, BM, and thymus of the mice were harvested for evaluation of T1D, insulitis, and chimerism levels. As shown in Fig. 1a, 100 days after BMT, 68% and 57% of the CD3/CD8 conditioned control mice and the MHC-matched BMT mice developed T1D, respectively. In contrast, none of the MHC-mismatched BMT mice developed T1D. Furthermore, 89% and 72% of the residual islets in the control mice and the MHC-matched BMT mice had insulitis, respectively (Fig. 1b). However, none of the islets in the MHC-mismatched BMT mice had insulitis, although a small portion of them showed peri-insulitis (Fig. 1b).

We then evaluated chimerism levels in the recipients at the end of experiments (on day 100 after BMT). There were no significant differences in the chimerism levels between the MHC-matched and MHC-mismatched BMT mice. In both groups, the spleens contained 30–40% host-type TCR^+^ T cells and B220^+^ B cells (Fig. 1c, isotype controls in this figure and other figures are shown in Additional file 1: Figure S1), and the BM contained 45–49% host-type B220^+^ B cells (Fig. 1d). De novo developed CD4^+^ CD8^+^ DP thymocytes also existed in both MHC-matched and MHC-mismatched BMT mice (Fig. 1e). However, the percentage of host-type DP thymocytes in the MHC-mismatched BMT mice was three to fourfold lower than that in MHC-matched mice (Fig. 1e), indicating negative selection occurred in the MHC-mismatched BMT mice.

Taken together, our results confirmed the previous reports that mixed chimerism was present in the NOD mice conditioned with anti-CD3/CD8 Abs and followed by either MHC-matched or MHC-mismatched BMT. Induction of mixed chimerism with MHC-mismatched but not matched BM transplants prevents insulitis and T1D development in these mice.

### Transplantation of MHC-mismatched mESC-TEPs prevents insulitis and T1D development in the MHC-matched BMT NOD mice

We then determined whether transplantation of MHC-mismatched mESC-TEPs could prevent the development of insulitis and T1D in NOD mice. To this end, NOD mice were conditioned with anti-CD3 and anti-CD8 Abs and injected i.v. with BM and CD4^+^ T cell-depleted spleen cells (20 × 10^6^ each) from MHC-matched H-2^g7^ B6 mice as described above. Groups of the BMT recipient were also injected i.t. with MHC-mismatched B6 mESC-TEPs (EpCAM1^+^ cells) or mESC-control cells (EpCAM1^−^ cells).

**Fig. 2:**
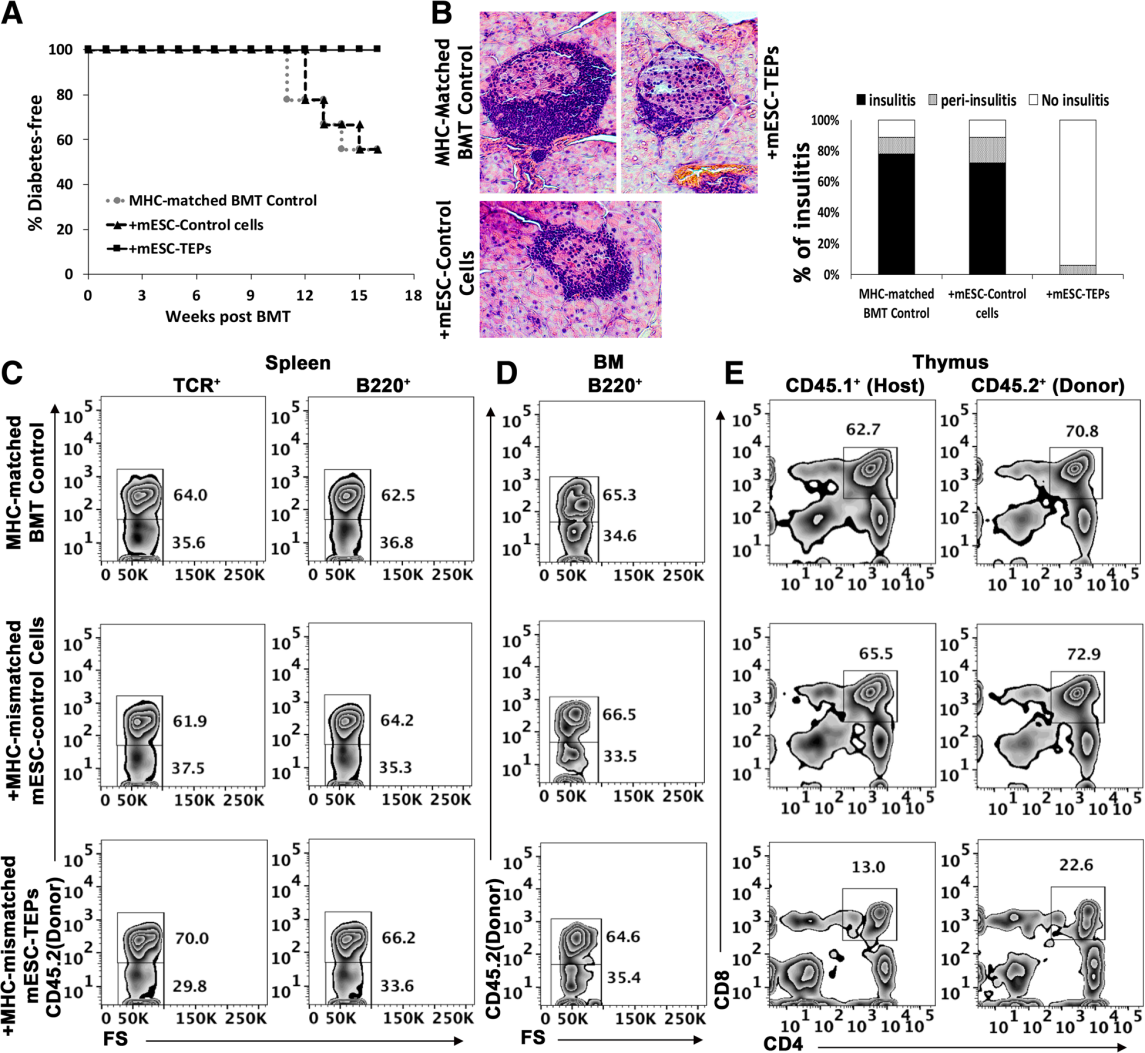
Transplantation of MHC-mismatched mESC-TEPs prevents insulitis and T1D development in the MHC-matched BMT recipients. Wild-type NOD mice were injected i.v. with anti-CD3/CD8 Abs on days − 8 and − 3 as in Fig. 1. On day 0, the conditioned mice were injected i.v. with BM and CD4^+^ T cell-depleted spleen cells (20 × 10^6^ each) from MHC-matched H-2^g7^ B6 mice. Groups of the BMT recipient were also injected i.t. with MHC-mismatched B6 mESC-EpCAM1^+^ TEPs (5 × 10^4^) or mESC-EpCAM1^−^ control cells (5 × 10^4^). Diabetes development was monitored weekly by blood glucose analysis. On day 100, the pancreas, spleen, BM, and thymus were harvested from the mice. **a** Diabetes development curve after BMT. **b** Representative sections of H&E staining, and statistical analysis of the percentages of insulitis (right bottom). **a**, **b** The data are pooled from three independent experiments (4–5 mice per group in each experiment). **c** One representative FACS profile of spleen cells for the percentages of donor (CD45.2^+^) or host (CD45.2^−^) T cells (TCR^+^) and B cells (B220^+^). **d** One representative FACS profile of BM cells for the percentages of donor (CD45.2^+^) or host (CD45.2^−^) B cells (B220^+^). **e** Gated donor (CD45.2^+^) or host (CD45.1^+^) thymocytes were shown in CD4 versus CD8. The percentages of CD4^+^CD8^+^ DP thymocytes are shown

The mice were monitored for T1D development. On day 100, the pancreas, spleen, BM, and thymus were harvested from the mice for evaluation of T1D, insulitis, and chimerism levels. As shown in Fig. 2a, 100 days after BMT, 53% of the MHC-matched BMT control mice developed T1D. Transplantation of mESC-derived control cells (EpCAM1^−^ cells) did not reduce the incidence of T1D. In contrast, transplantation of mESC-TEPs prevented the development of T1D with none of the mice having T1D. Furthermore, 78% and 73% of the residual islets in the MHC-matched BMT control mice or mESC-control cell-transplanted mice had insulitis, respectively (Fig. 2b). In contrast, none of the islets in the mESC-TEP-transplanted mice had insulitis, although a small portion of them showed minor peri-insulitis (Fig. 2b).

We then evaluated chimerism levels in the BMT recipients. Again, there were no significant differences in the chimerism levels among the 3 groups, and the spleens in each group contained 29–40% host-type TCR^+^ T cells and B220^+^ B cells (Fig. 2c). The BM contained similar percentages of host-type B220^+^ B-cells (Fig. 2d). Host CD4^+^CD8^+^ DP thymocytes also de novo developed in all of the mice (Fig. 1e). Similar to the MHC-mismatched BMT recipients, the percentage of donor and host DP thymocytes in the mESC-TEP-transplanted mice was three to fivefold lower than that in MHC-matched BMT control mice or mESC-control cell-transplanted mice, suggesting negative selection occurred in the mESC-TEP-transplanted mice.

Taken together, our results suggest that transplantation of MHC-mismatched mESC-TEPs results in the prevention of insulitis and T1D development in MHC-matched BMT mice, which is due, at least partly, to the deletion of autoreactive host T cells in the thymus.

### Transplantation of MHC-mismatched ESC-TEPs into the MHC-matched BMT recipients leads to an increased number and function of Tregs

**Fig. 3:**
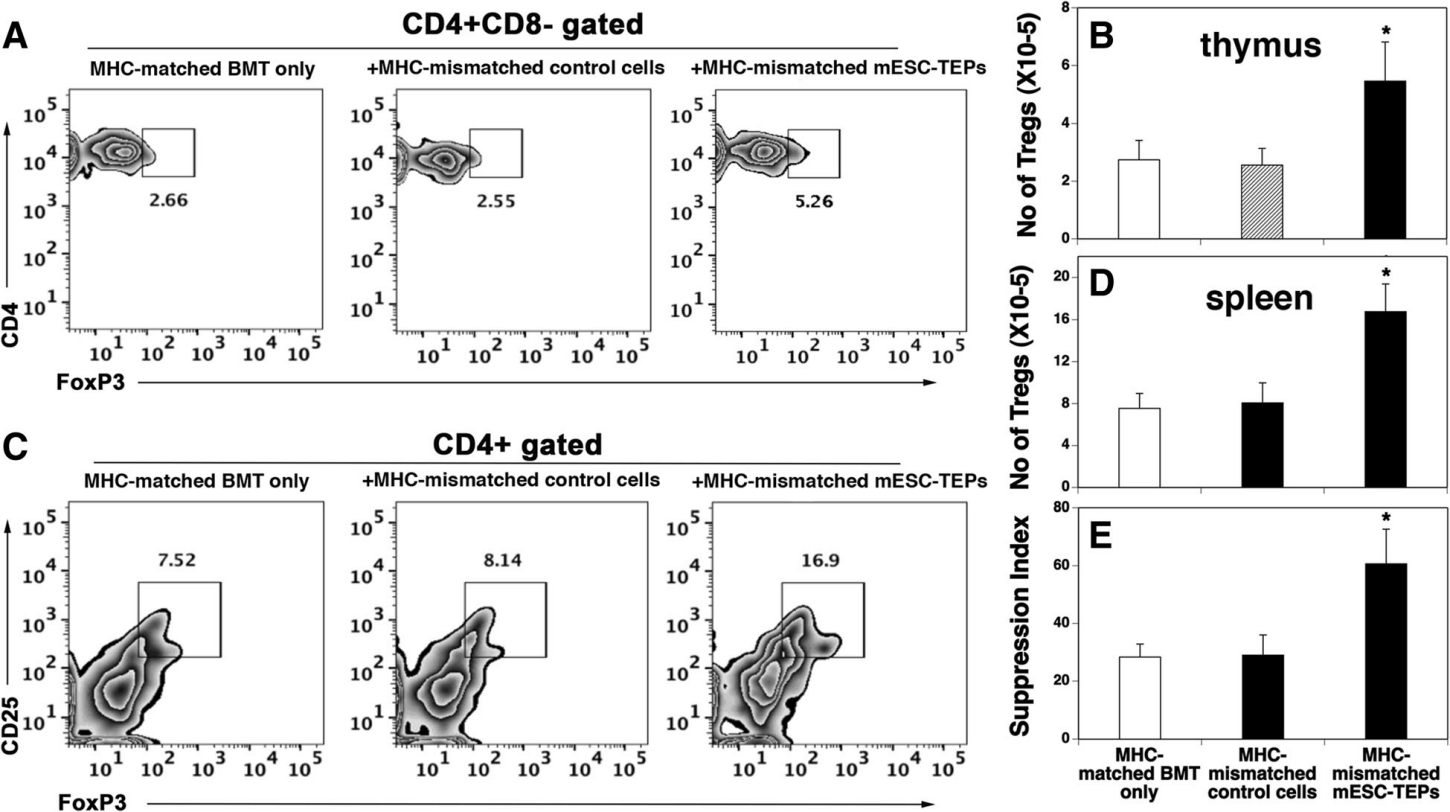
mESC-TEP-treated recipients have an increased number and function of Tregs. NOD mice were injected i.v. with anti-CD3/CD8 Abs on days − 8 and − 3. On day 0, the conditioned mice were injected i.v. with BM and CD4^+^ T cell-depleted spleen cells from MHC-matched H-2^g7^ B6 mice. Groups of the BMT recipient were also injected i.t. with MHC-mismatched B6 mESC- EpCAM1^+^ TEPs or mESC-control cells (EpCAM1^−^ cells) as in Fig. 2. On day 100, Tregs in the thymus and spleen were analyzed by flow cytometry. **a** Representative flow cytometric profiles showing thymic Tregs. **b** The number of thymic CD4^+^CD8^−^CD3^+^FoxP3^+^ Tregs from each group. **c** Representative flow cytometric profiles showing the Tregs in the spleen of the recipients. **d** The number of splenic CD4^+^CD25^+^FoxP3^+^ Tregs from each group. **e** CD4^+^CD25^+^ Tregs were purified from the spleen of each group. CD4^+^CD25^−^ Teffs were purified from wild-type NOD mice. The Teffs were stimulated with anti-CD3 antibody (0.5 μg/ml) in the presence or absence of Tregs at a ratio 2:1 for 3 days. The proliferation of Teffs was measured by [^3^H] thymidine incorporation. The results are presented as suppression index. **c**–**e** The data are expressed as mean ± SD from one of three independent experiments with similar results (4–5 mice per group in each experiment). *P < 0.05, compared with mice given MHC-matched BMT only

Accumulating data have shown that Tregs play an important role in controlling T1D [3, 39]. Since TECs can support Treg development, we analyzed Tregs in the NOD mice that had been subjected to MHC-matched BMT alone, or MHC-matched BMT plus MHC-mismatched EpCAM1^+^ mESC-TEPs or mESC- EpCAM1^−^ control cells as shown in Fig. 2. As shown in Fig. 3a–d, the number of Tregs in the thymus and spleen of mESC-TEP-treated recipients were two to threefold higher than that in MHC-matched BMT control mice or mESC- EpCAM1^−^ control cell-transplanted mice. It is likely that the increased number of Tregs is also responsible for the prevention of diabetes in mESC-TEP-transplanted NOD mice.

To determine whether the Tregs were functional, we evaluated the ability of the Tregs to inhibit the proliferation of Teffs from NOD mice. CD4^+^CD25^+^ Tregs were purified from the spleen of MHC-matched BMT control mice, mESC- EpCAM1^−^ control cell-, or mESC- EpCAM1^+^ TEP-transplanted mice. CD4^+^CD25^−^ Teffs were purified from wild-type NOD mice. The Teffs were stimulated with anti-CD3 antibody in vitro in the presence or absence of the Tregs. Cell proliferation was determined by [^3^H] thymidine incorporation 3 days later. As shown in Fig. 3e, Tregs from mESC-TEP-treated recipients had about twofold higher activity than those from control mice in inhibiting the proliferation of Teffs. The data indicate that the Tregs from mESC-TEP-treated recipients were functional.

### MHC-mismatched mESC-derived TECs express higher level of proinsulin than host TECs

**Fig. 4:**
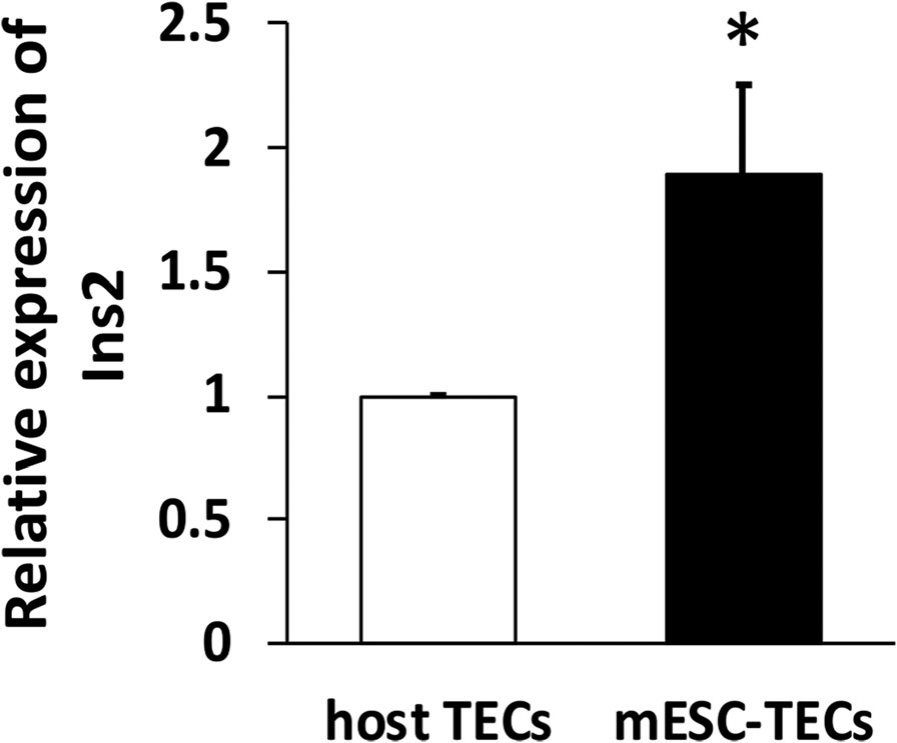
mESC-TECs express higher levels of proinsulin than host TECs. Anti-CD3/CD8 conditioned NOD mice were injected i.v. with BM and CD4^+^ T cell-depleted spleen cells from MHC-matched H-2^g7^ B6 mice and i.t. with GFP^+^ B6 mESC- EpCAM1^+^ TEPs as in Fig. 2. On day 100, the thymi were harvested from the mice, and mESC-TECs (GFP^+^ CD45^−^ EpCAM^+^) or host TECs (GFP^−^ CD45^−^ EpCAM^+^) were isolated. The expression of Ins2 in the mESC-TECs and host TECs was analyzed by qRT-PCR. Expression levels for each gene were normalized to the housekeeping gene GAPDH and are presented as relative expression compared with host TECs. Data show one of three independent experiments with similar results

Many studies have shown that insulin/proinsulin is the key autoantigen in initiating the autoimmunity of T1D [23–25]. Proinsulin is the pre-form of insulin. Mice have two proinsulin genes, Ins1 and Ins 2. Ins1 is exclusively expressed in the pancreas and Ins 2 is expressed in both thymus and pancreas [40]. We have previously reported that most of mESC-TEPs develop into cTECs (CD45^−^EpCAM^+^Ly51^+^) and mTECs (CD45^−^EpCAM^+^Ly51^−^) in vivo [38]. We analyzed the expression levels of Ins 2 in total TECs (CD45^−^EpCAM^+^) by qRT-PCR. By using GFP^+^ B6 mESCs to separate mESC-TECs (GFP^+^ CD45^−^ EpCAM^+^) and host TECs (GFP^−^ CD45^−^ EpCAM^+^), we found that the expression level of Ins2 in mESC-TECs was about twofold higher than that in host TECs (Fig. 4).

### Transplantation of MHC-mismatched mESC-TEPs does not lead to an upregulation of PD-1 and downregulation of IL-7Rα on host-type peripheral T cells

**Fig. 5:**
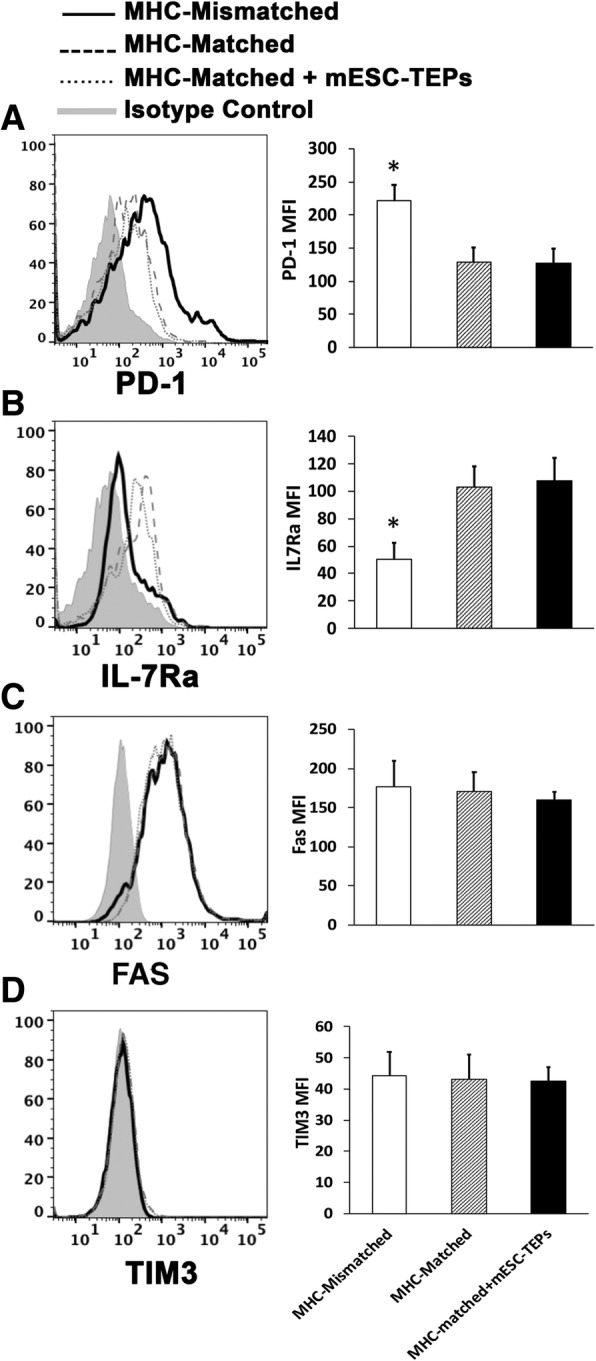
MHC-mismatched mESC-TEP-transplanted mice do not have an upregulated PD-1 and downregulated IL-7Rα expression on host-type peripheral T cells. Wild-type NOD mice were injected i.v. with anti-CD3/CD8 Abs on days − 8 and − 3 as in Fig. 1. On day 0, the conditioned mice were injected i.v. with BM and CD4^+^ T cell-depleted spleen cells (20 × 10^6^ each) from MHC-matched H-2^g7^ B6 mice or MHC-mismatched B6 mice as in Fig. 1. A group of the MHC-matched BMT recipients was also injected i.t. with B6 mESC-EpCAM1^+^ TEPs (5 × 10^4^) as in Fig. 2. On day 100, the expression of PD-1, IL-7Rα, FAS and TIM3 by residual splenic host-type T cells (CD45.1^+^CD4^+^TCRβ^+^) was analyzed by flow cytometry. The Expression levels of **a** PD-1, **b** IL-7Rα, **c** FAS and **d** TIM3. One representative FACS profile and mean fluorescence (*n* = 4–6) are shown. *P < 0.05, vs MHC-matched BM transplants

It has been reported that, in MHC-mismatched BM-transplanted NOD mice, the residual host-type peripheral T cells were anergic caused by the upregulation of PD-1 and downregulation of IL-7Rα [21]. We determined whether this also occurred in the mESC-TEP-transplanted NOD recipients. Consistent with a previous report, the expression of PD-1 was about twofold increased and the expression of and IL-7Rα was about twofold decreased on the host-type T cells of MHC-mismatched BM-transplanted NOD mice, as compared with those in MHC-matched BM-transplanted mice (Fig. 5). However, the expression of PD-1 and IL-7Rα was not increased or decreased on the host-type T cells of ESC-TEP-transplanted mice. We also analyzed the expression of FAS and TIM3, molecules related to apoptosis and T cell exhaustion, and did not find a significant difference among the groups. Therefore, the peripheral tolerance mechanisms induced by MHC-mismatched BM transplants and mESC-TEPs appear to be different.

## Discussion

In this study, we demonstrate that induction of mixed chimerism with MHC-matched BM transplants from nonautoimmune donors cannot prevent the development of insulitis and T1D in NOD mice, consistent with other reports [19, 21]. In contrast, transplantation of MHC-mismatched mESC-TEPs prevents insulitis and T1D development in MHC-matched BMT NOD mice. Although induction of mixed chimerism with MHC-mismatched BM transplants also prevents the development of insulitis and T1D in NOD mice, the underlying mechanisms appear to be different from those from the transplantation of MHC-mismatched mESC-TEPs.

It has been reported that NOD or T1D patients have a defect in central tolerance [9–12, 41]. Transplantation of MHC-mismatched BM cells or MHC-mismatched mESC-TEPs, at least partly, corrected the negative selection, resulting in the deletion of autoreactive T cells in the thymus. However, the former was mediated by donor DCs [19], whereas the latter was mediated by mESC-TECs.

The autoimmunity in NOD mice is related to the I-A^g7^ [7, 8]. It has been shown that the I-A^g7^ has a weak and unstable peptide-binding property, leading to the defect in thymic negative selection [9, 10]. Conversely, transgenic NOD mice that express MHC class II genes other than I-A^g7^ prevent T1D development [13, 14]. The prevention of diabetes in the MHC-mismatched B6 mESC-TEP-transplanted NOD mice is probably, at least partly, due to introducing protective MHC molecules in TECs.

We have also shown that mESC-derived TECs expressed a higher level of Ins2 than host TECs in the NOD mice. Although multiple inlet autoantigens have been implicated in T1D, insulin/proinsulin is a major autoantigen associated with T1D [23–25]. It has been shown that thymic insulin levels play a critical role in insulin-specific T cell self-tolerance. Lower levels of insulin expression in the thymus are associated with the generation of autoreactive T cells and the development of T1D [1, 40, 42]. Therefore, the higher expression of proinsulin in the mESC-TECs may also contribute to the restoration of defective negative selection in NOD mice.

In addition to the deletion of autoreactive T cells in the thymus, peripheral tolerance also plays a role in the prevention of insulitis and T1D. Our data suggest that the mechanisms of the peripheral tolerance induced by the two approaches seem also to be different. In the MHC-mismatched BMT mice, the residual host-type peripheral T cells were anergic due to upregulation of PD-1 and downregulation of IL-7Rα. In contrast, transplantation of mESC-TEPs did not lead to the upregulation of PD-1 and downregulation of IL-7Rα. Instead, it resulted in an enhanced Treg development in the thymus, leading to an increased number of Tregs in the periphery.

Reduced frequency and/or function of Tregs in both NOD mice and T1D patients were observed by some groups [3, 39], although these results were disputed by others [43, 44]. The importance of Tregs in controlling T1D has been clearly established [3, 45]. For example, NOD mice with Treg defect have an accelerated T1D [46–49]. It has also been shown that the proportion of Tregs in the pancreatic islets progressively decreases with inflammation [50]. We have demonstrated that transplantation of mESC-TEPs leads to an increased number of Tregs that can inhibit the proliferation of Teffs in NOD mice. Our data suggest that the increased number of Tregs plays a role in the prevention of T1D. Although it has been reported that other stem cell technology, such as ESC- or induced pluripotent stem cell (iPSC)-derived pancreatic progenitors and allogeneic hematopoietic stem cell transplant have the potential for curing T1D [51, 52], the mechanisms by which our ESC-TECs ameliorate T1D appear to differ from these approaches.

In this study, we have used mESCs to show the feasibility of ESC-TEPs/TECs to ameliorate insulitis and T1D in NOD mice. Using mESC-derived cells also facilitates mechanistic studies. In the future, we will also test the ability of human ESC- and iPSC-derived TEPs/TECs to prevent and treat T1D. Our present studies using NOD mice have some limitations. For example, we transplanted mESC-TEPs into mice prior to the onset of insulitis. Therefore, this protocol was designed to prevent T1D. In future studies, we will determine the ability of ESC- and iPSC-TEPs/TECs to reverse or treat T1D by transplantation of the cells after the onset of insulitis.

## Conclusions

In summary, we have demonstrated that transplantation of MHC-mismatched mESC-TEPs prevents insulitis and T1D development in NOD mice. This is likely due to the restoration of defects in both central and peripheral tolerance in NOD mice. Our findings justify further evaluation of human ESC- and iPSC-derived TEPs/TECs in the prevention and treatment of T1D in both animals and human patients.

## Additional file

Additional file 1: **Figure S1.** Isotype antibody staining for Figs. 1, 2, and 3. (PDF 205 kb)

## Data Availability

The datasets used and/or analyzed during the current study available from the corresponding author on reasonable request.

## Abbreviations

Abs: Antibodies
BM: Bone marrow
BMT: BM transplantation
DE: Definitive endoderm
EAE: Experimental autoimmune encephalomyelitis
GVHD: Graft-versus-host disease
HLA: Human leukocyte antigen
Ins2: Proinsulin 2
k: Keratin
mESCs: Mouse embryonic stem cells
MHC: Major histocompatibility complex
MOG: Myelin oligodendrocyte glycoprotein
qRT-PCR: Real-time quantitative reverse-transcription and polymerase chain reactions
T1D: Type 1 diabetes
TECs: Thymic epithelial cells
TEPs: Thymic epithelial progenitors
Tregs: Regulatory T cells
