# Supplementary figures and images for: Transplantation of MHC-mismatched mouse embryonic stem cell-derived thymic epithelial progenitors and MHC-matched bone marrow prevents autoimmune diabetes
Source: Stem Cell Res Ther. 2019 Aug 6;10:239. doi: 10.1186/s13287-019-1347-1 (PMC6685174; doi:10.1186/s13287-019-1347-1)

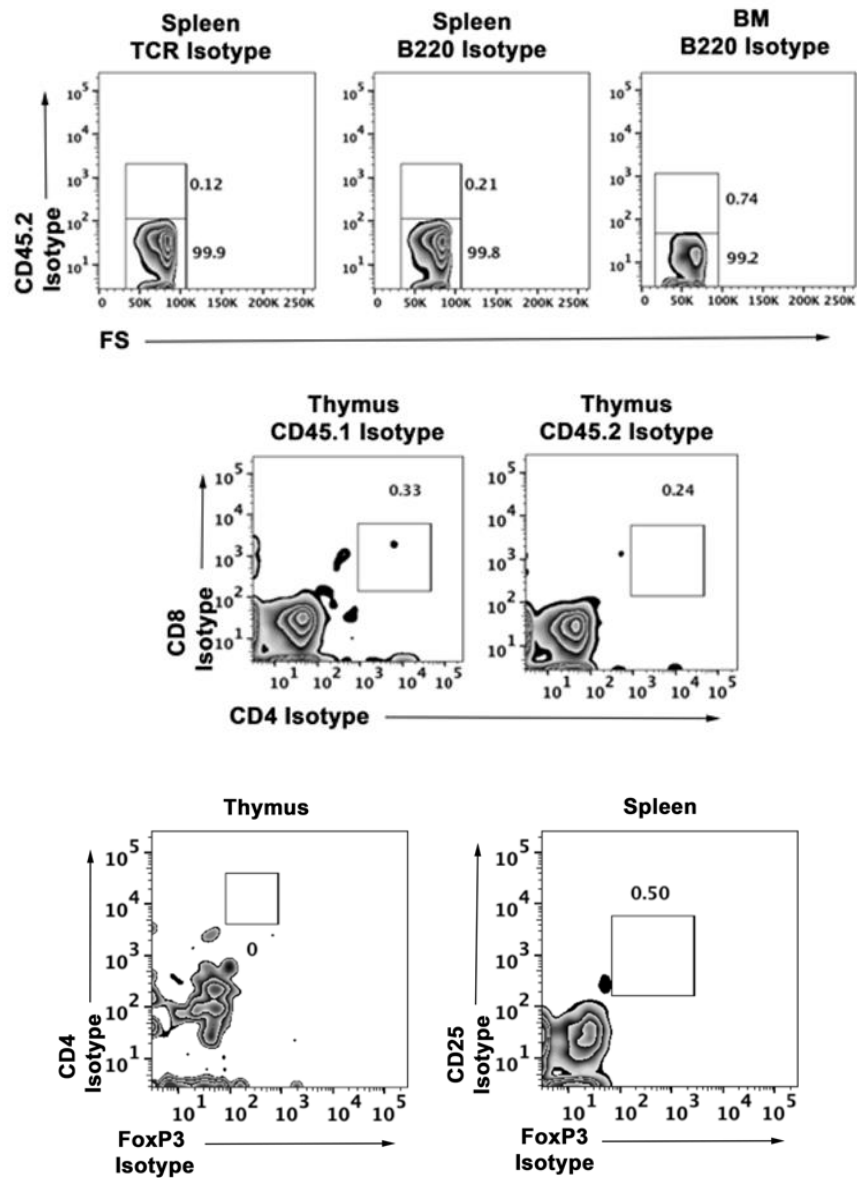

Supplemental Figure 1. Isotype antibody staining for Figures 1, 2 and 3.

Supplement: Supplementary file 1 — Figure S1. Isotype antibody staining for Figs. 1, 2, and 3. (PDF 205 kb) [file 13287_2019_1347_MOESM1_ESM.pdf]
